# Characterization of a reversible thermally-actuated polymer-valve: A potential dynamic treatment for congenital diaphragmatic hernia

**DOI:** 10.1371/journal.pone.0209855

**Published:** 2018-12-27

**Authors:** Justin S. Baba, Timothy E. McKnight, M. Nance Ericson, Anthony Johnson, Kenneth J. Moise, Boyd M. Evans

**Affiliations:** 1 Electrical and Electronics Systems Research Division, Oak Ridge National Laboratory, Oak Ridge, Tennessee, United States of America; 2 Biophotonics Center, Department of Biomedical Engineering, Vanderbilt University, Nashville, Tennessee, United States of America; 3 Department of Obstetrics, Gynecology, Baylor College of Medicine, Houston, Texas, United States of America; Max-Planck-Institut fur Kolloid und Grenzflachenforschung, GERMANY

## Abstract

**Background:**

Congenital diaphragmatic hernia (CDH) is a fetal defect comprising an incomplete diaphragm and the herniation of abdominal organs into the chest cavity that interfere with fetal pulmonary development. Though the most promising treatment for CDH is via interventional fetoscopic tracheal occlusion (TO) surgery *in-utero*, it has produced mixed results due to the *static* nature of the inserted occlusion. We hypothesize that a suitable noninvasively-actuatable, cyclic-release tracheal occlusion device can be developed to enable *dynamic* tracheal occlusion (dTO) implementation.

**Objective:**

To conduct an *in-vitro* proof-of-concept investigation of the construction of thermo-responsive polymer valves designed for targeted activation within a physiologically realizable temperature range as a first step towards potential development of a noninvasively-actuatable implantable device to facilitate *dynamic* tracheal occlusion (dTO) therapy.

**Methods:**

Six thermo-responsive polymer valves, with a critical solution temperature slightly higher than normal physiological body temperature of 37°C, were fabricated using a copolymer of n-isopropylacrylamide (NIPAM) and dimethylacrylamide (DMAA). Three of the valves underwent ethylene oxide (EtO) sterilization while the other three served as controls for EtO-processing compatibility testing. Thermal response actuation of the valves and their steady-state flow performances were evaluated using water and caprine amniotic fluid.

**Results:**

All six valves consisting of 0.3-mole fraction of DMAA were tested for thermal actuation of caprine amniotic fluid flow at temperatures ranging from 30–44°C. They all exhibited initiation of valve actuation opening at ~40°C with full completion at ~44°C. The overall average coefficient of variation (CV) for the day-to-day flow performance of the valves tested was less than 12%. Based on a Student t-test, there was no significant difference in the operational characteristics for the EtO processed versus the non-EtO processed valves tested.

**Conclusions:**

We successfully fabricated and demonstrated physiological realizable temperature range operation of thermo-responsive polymer valves *in-vitro* and their suitability for standard EtO sterilization processing, a prerequisite for future *in-vivo* surgical implantation testing.

## Introduction

Congenital diaphragmatic hernia (CDH) is a fetal defect involving absence of portions of the diaphragm and herniation of abdominal organs into the chest that interfere with fetal pulmonary development. The incidence of this congenital anomaly is approximately 1 in 1754–5882 births with regional, ethnic, and gender variations [1–8]. A reported range of 40–80% of infants diagnosed with severe CDH die at birth or shortly thereafter [8–10]. In a review of the Healthcare Cost and Utilization of Project 2003 Kids’ Inpatient Database (KID), CDH ranked among the most costly and lethal of congenital malformations. CDH was sixth for average length of stay at 25 days (95% CI: 23.1–26.8 days) with total annual patient care costs of $179,470,456 (95% CI: $156,501,285 –$202,439,627). Excluding anencephaly and trisomies 13 and 18, CDH was associated with the highest risk of in-hospital neonatal death (34.4%) [11]. More recent adjusted estimates peg CDH costs at $250,000 per case with a US annual cost that exceeds $230 million, and the data reveal that earlier interventions lead to reduced overall patient treatment costs [12].

Initial treatments for CDH were based on attempts to repair herniated fetal diaphragms pre-birth via open fetoscopic surgery. Those efforts were fraught with significant risk to both mother and child, thus yielding a perinatal mortality rate in excess of 70% [13]. Subsequently, an alternative treatment method emerged based on clinical observations that non-CDH infants born with a condition of tracheal blockage–such as laryngeal or tracheal atresia–manifested enlarged hyperplastic lungs [14]. Consequently, intentional fetoscopic tracheal occlusion (TO) was evaluated as an *in-utero* bridging procedure for facilitating proper lung development. The interventive procedure yields an accumulation of pulmonary fluid that facilitates lung enlargement, which in-turn serves to minimize or reverse abdominal organ advancement into the pulmonary cavity [15,16]. Following those reports, there was a “PLUG” (plug-lung-until-it-grows) odyssey, with many different experimental manipulations to restrict egress of fetal lung liquid that included the use of cuffs, polymeric foams, magnetic valves, umbrellas, surgical dissection and detachable vascular occlusive balloons [17].

The standard technique for fetal endoluminal tracheal occlusion (FETO) utilizes a single port for fetoscopic access to the trachea at 26–30 weeks gestation. Under direct ultrasound visualization, a detachable balloon is advanced just above the fetal carina and inflated with isotonic solution. Once secured in place, the *static* obstruction remains until surgical removal at approximately 34 weeks gestation. Though now obsolete, the primary device utilized, the GVB16 Goldvalve detachable balloon, “was originally designed for interventional radiological procedures to occlude anomalous vessels” [18]. It had a maximum volume of 0.8-ml, diameter of 8.0-mm and length of 21.0-mm and was implemented to provide complete, *static* obstruction in the fetal trachea until its removal. Currently, the Goldballoon is the detachable balloon device being utilized in ongoing research evaluating the potential benefits of FETO for the treatment of CDH [19]. This particular device, from BALT Extrusion, was designed for endovascular treatment by interventional neuroradiology for intracranial vascular pathologies [20,21]. At present, the device is not commercially available in the United States. Clearly, none of the aforementioned FETO devices were specifically designed for *in-utero* treatment of CDH.

Initial studies of TO have revealed provisional benefits in CDH that include both an increase in lung tissue stretch and accelerated lung growth volume. However, prolonged *static* TO has also revealed deleterious effects. They include an increase in alveolar wall thickness, a reduction in surfactant synthesis due to increased differentiation of type II into type I pneumocytes, and ultimately respiratory failure after birth [22–26]. Early release of *static* TO has been attempted to improve surfactant production in fetal lambs with and without surgically-induced CDH. It has produced varied responses in the preservation of type II pneumocytes. Adversely, early release has resulted in loss of the positive gains in lung maturation, growth, and vascular remodeling associated with TO [26–28]. In recent studies of catheterized fetal lambs, Nelson *et al*. [29] have demonstrated that cyclic release of TO, late in gestation, induced optimal lung growth with morphological maturation of hypoplastic lung parenchyma whilst maintaining a population of type II pneumocytes. Overall, these findings indicate that TO continues to have promising therapeutic potential for CDH.

Before the benefits of TO can be fully realized, it is critically important to develop physiologic methodologies whereby the TO is not a *static* blockage, but rather a *dynamic* one that can be clinically manipulated cyclically over the entire gestational duration [30]. Recently, Jelin *et al*. [31] reported on the construction and demonstration of a novel *dynamic* tracheal occlusion (dTO) device that operates based on a built-in pressure-sensitive valve. The valve actuated at a preset pressure differential of 80-mmH_2_O to allow for *dynamic* lung fluid efflux. In a fetal lamb CDH model, the fetoscopic implanted dTO device facilitated improvements in lung morphometrics and in functional outcomes. Their design, though promising, is disadvantaged by its pre-implantation, fixed pressure set-point that may not be optimal for achieving ideal lung morphometrics and functional outcomes over the entire gestation period. Consequently, we report on the development of a more flexible-control dTO implant device that can be manipulated non-invasively to provide for remote, clinical control over fetal lung volume efflux based on adjustable time-period-modulation of valve actuation. This is a significant improvement over the aforementioned pre-implantation, fixed pressure set-point actuation dTO design.

Accordingly, the focus of this communication is the *in-vitro* proof-of-concept demonstration of the construction of thermo-responsive polymer valves designed for targeted activation within a physiologically realizable temperature range. Accomplishing this objective is the first step towards achieving the aforementioned dTO implant device. The ensuing summary is provided to help contextualize future clinical implementation of the fully envisioned dTO implant device system that is still under development.

Our novel dTO system is comprised of a balloon occlusion, styled after the Goldvalve device [32,33], that also features an internal lumen as a path for pulmonary fluid flow. A wireless, RF-responsive microvalve element is inserted into this lumen. The microvalve element consists of a thermally-actuated polymer-valve body with an embedded resistive heater and a RF coil-driving unit. The RF unit provides clinically directed inductive power to the heating element at very specific RF frequencies. At normal physiological temperature (37°C), the polymer-valve body remains in a swollen state that fills up the balloon lumen, thus occluding pulmonary fluid flow and providing for accumulation of fluid to yield the desired expansion of the fetal lung. Under specific RF excitation, the embedded valve-heater increases the temperature of the polymer by 3–5°C. This results in a reversible, thermal collapse of the polymer-valve body yielding a principle annular flow of pulmonary fluid through the balloon lumen via around its shrunken cross-section. Continuous flow is maintained during RF excitation and for a short period afterwards. Thereafter, the polymer-valve body returns to normal physiological temperature accompanied by reversible swelling that re-occludes the balloon lumen cross-section [34,35].

## Materials and methods

### Materials

N-isopropylacrylamide (NIPAM), dimethylacrylamide (DMAA), 3-methacryloxypropyltri-methoxysilane (MAPTOS), N-N’-methylenebisacrylamide (MBAA), ammonium persulfate (APS), ethanol, acetic acid, and tetramethylethylenediamine (TEMED) were purchased from Sigma-Aldrich (St. Louis, MO, USA) and used as received. Though the native monomer constituents of these chemicals are cytotoxic, their bioavailability and cytotoxicity are substantially reduced upon polymerization [36,37]. Pertinently, the US Food and Drug Administration (FDA) has approved some formulations of polyacrylamide for human and animal consumption, and also for human applications that include denture adhesives [37].

Phosphate Buffered Saline (PBS) was obtained from ATCC (Manassas, VA, USA) and used as received. Borosilicate glass capillaries were obtained from Sutter Instruments (Novato, CA, USA) and ultrasonically cleaned, using a Branson Ultrasonic Bath Cleaner: Model 1510 (Branson Ultrasonics Corporation, Danbury, CT, USA), with detergent and water prior to use.

### Polymer-valve fabrication

Polymer valves were grafted onto the surface of a heater core comprising a glass capillary tube treated with a silane-coupling agent, as described in the following details.

First, glass capillary tube heater cores of 0.5-mm inner diameter (i.d.) and 1.0-mm outer diameter (o.d.) were cut to size (15-mm length). Next, they were ultrasonically cleaned (Branson: Model 1510) using a strong soap solution followed by copious rinsing in distilled water. The heater cores were then dried and submersed in a solution comprised of 1 mL of MAPTOS in 200 mL of ethanol, into which 6 mL of dilute acetic acid (1:10 glacial acetic acid in water) was added just before use. The glass heater cores were placed in this solution for 2 minutes, removed and rinsed extensively with ethanol, then air-dried. All subsequent fabrication steps were then conducted under a nitrogen-purged environment.

The MAPTOS-treated glass cores were placed individually within 10-mm long, cleaned glass capillary tubes (1.78-mm i.d., 2.0-mm o.d.) that had not been MAPTOS-treated, such that a short section of the internal core emerged from each end of the untreated tubes. The outer tube served as a mold for subsequent polymerization reactions. A polymerization solution comprising 200-μL of NIPAM in phosphate buffered saline (50-mg/mL), 2-μL of MBAA (50 mg/mL in water) and various amounts of DMAA (4, 6, 8, or 10-μL) was then mixed with 20-μL of APS (50-mg/mL in water) and 20-μL of TEMED (25 μL/mL in water). A portion of this reaction mixture was then quickly dispensed onto an emergent end of each glass core assembly. The amount used was enough to fill the annular gap between the MAPTOS-treated heater core and the inner diameter of the surrounding untreated glass tube serving as the mold for the subsequent polymerization. The filled molds were reacted overnight under nitrogen purge within a 100% relative humidity environment; typically, this was for 17 hrs.

Next, the polymerized valve molds were removed from nitrogen purge and iteratively immersed into hot (~95°C) and then cool (~25°C) water baths to induce, respectively, shrinkage and swelling of the polymer valves. The thermal cycling process resulted in detachment of the polymer from the mold, thus enabling the complete release of the heater-core-grafted-polymer-valve (henceforth referenced as polymer-valve) unit from its exterior mold. Following this, the released polymer valves were extensively rinsed in a continuously stirred water bath to wash out residual reagents. The thermal cycling and post-wash treatment was extended and iterated to flush any residual monomers from the fabricated devices. Afterwards, the polymer valves were either tested immediately or inserted into the lumen of a balloon implant for later testing.

## Results and discussion

**Fig 1:**
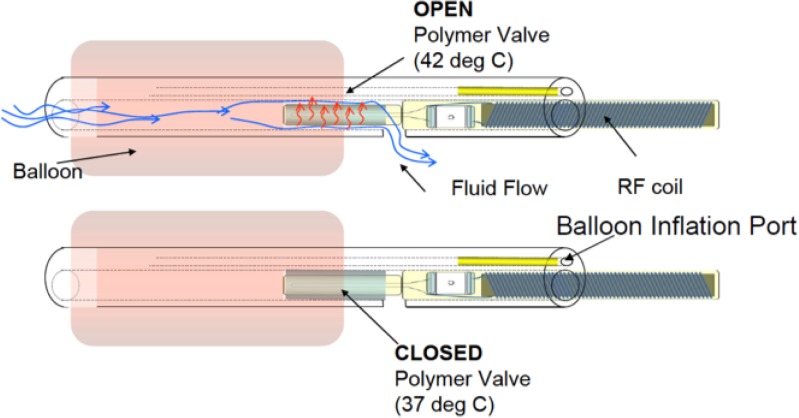
Schematic representation of the *dynamic* tracheal occlusion valve. It comprises a saline filled detachable balloon that obstructs the fetal trachea and provides an internal flow path via an RF actuatable thermo-responsive polymer-valve.

Responsive polymer micro-valves have been reported extensively in the literature. They enable reversible valving of molecular transport and fluid flow using polymer response to temperature, pH, or electrochemical redox [38–48]. Though thermo-responsive polymer valves are not new, the application to the development of a clinically actuatable *dynamic* tracheal occlusion (dTO) valve is indeed novel. This is substantiated by two issued patents [34,35]. Our approach, utilizing thermo-responsive polymer microvalving, is depicted in Fig 1. The framework for the system is a *static* balloon occlusion, manufactured by NuMED, Inc. (Hopkinton, NY, USA), featuring a 1.6-mm i.d. lumen flow path. This detachable balloon may be catheter deployed using conventional surgical tools for fetoscopic surgery and immobilized in place by inflation of the outer balloon component via infusion of physiological saline.

As described, the 1-mm o.d. glass heater core element inserted into the polymer micro-valve unit was covalently grafted with the thermo-responsive copolymer of NIPAM and DMAA. Variations of this polymer have been used to provide extremely high backpressures (>2500 psi) in the closed (<32°C) state of thermally-actuated, polymer-monolith valves [38]. Additionally, they have been used in sidewall functionalization of Si and SiO_2_ based microfluidic devices (sub 100-μm dimensions) for valving of localized flows and generation of 5-psi backpressure between the closed and open states [39]. For the current application, the polymer is designed to have a transition temperature slightly above normal physiological temperature in the range of 40–43°C. Operatively, the polymer remains in a swollen state (valve closed) at normal body temperature and shrinks to open the valve at safe elevated temperatures under the envisioned, clinically directed RF stimulus.

Sterilization of polymer-valve units is a prerequisite for *in-vivo* implantation studies. Therefore, the dTO system is designed to be compatible with ethylene oxide (EtO) sterilization. Because EtO sterilization could potentially impact the microvalving functionality of the polymer, its impact was investigated and the results included as part of this proof-of-concept demonstration. Essentially, the hypothesis tested was that EtO sterilization can be conducted on dehydrated valve assemblies and that the performance of the resultant sterilized polymer-valve units is maintained when they rehydrate, such as upon fetal implantation.

### Covalent grafting of copolymers onto a glass heater core

**Fig 2:**
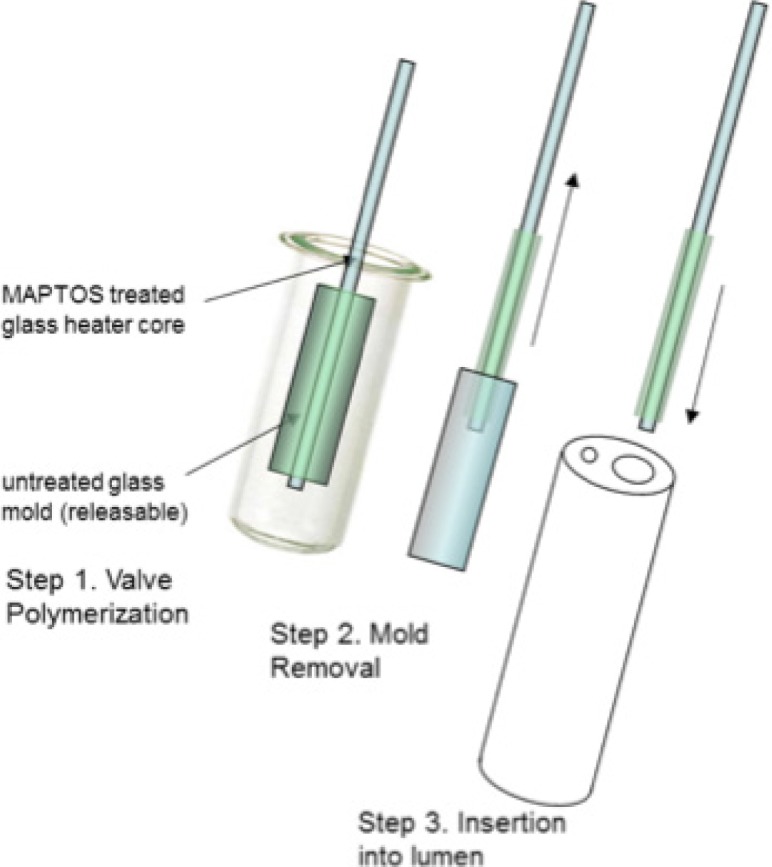
Fabrication steps of the thermally actuated polymeric valve.

Saitoh *et al*. [49] have previously reported on the construction of thermo-responsive flow control elements in microfluidic devices fabricated via covalent grafting of poly-n-isopropylacrylamide (pNIPAM) onto glass capillary tubes using MAPTOS (under the trade name of Bind-Silane) as a coupling reagent during APS-initiated and TEMED- accelerated polymerization. Similarly, our polymer-valve bodies were constructed by grafting the polymerization onto the outer surface of a MAPTOS-treated glass capillary and using a larger, untreated glass capillary as a mold (hereby referred to as glass mold). Note that the inner treated glass capillary also serves as the encapsulation for the RF-driven heating element, as depicted in Fig 1. Size control of the polymer grafted onto this element was achieved by first adjusting the length of the glass mold to the desired length of the polymer-valve body, as described. Subsequently, the heater core element was extended from the ends of the glass mold such that dispensed polymerization solution could be drawn in to fill the annular gap between the heater core and the outer glass mold via capillary action (Step 1, Fig 2).

As previously described, post overnight polymerization, separation of polymer-valve units from the glass mold assemblies was achieved by iterative immersion of the coupled parts into 95°C and 25°C water baths (Step 2, Fig 2). Likewise, thermal polymer-valve-body-collapse at elevated temperatures was utilized to enable the insertion of combined polymer-valve and heater-core elements into various assemblies for testing. Those included insertion into the lumen of the NuMed tracheal balloon (Step 3, Fig 2), or into clear glass or polymer-based capillary tubes for volumetric flow and valve actuation visualization studies. Note that heater-core system testing data is not included in this report but reserved for a future proof-of concept demonstration of the implementation of an externally applied RF signal to inductively drive the incorporated resistive heater for thermal microvalving actuation of the fabricated polymer valves.

### Optimization of thermal transition temperature of polymer-valve body

Shen *et al*. [50] have previously reported on the variation of thermal phase behavior of various copolymers of NIPAM formulated with acrylamide, DMAA, and hydroxyethyl-methacrylate. In their study, polymerizations were conducted at 60°C under a nitrogen purge using free radical polymerization with 2,2′-azobis (isobutyronitrile) (AIBN) and 3-mercaptopropionic acid (MPA) as an initiator and chain transfer reagent, respectively. Lower critical solution temperatures (LCSTs) of the various polymer formulations were determined in water based on the optical transmittance of pNIPAM-DMAA. LCSTs ranging from 36.9°C to 46.0°C were dependent upon the molar fraction of NIPAM that ranged from 0.686 to 0.893. Our approach differed from the aforementioned in employing the following: (1) ambient temperature polymerization, (2) APS as initiator, and (3) TEMED as accelerator.

**Fig 3:**
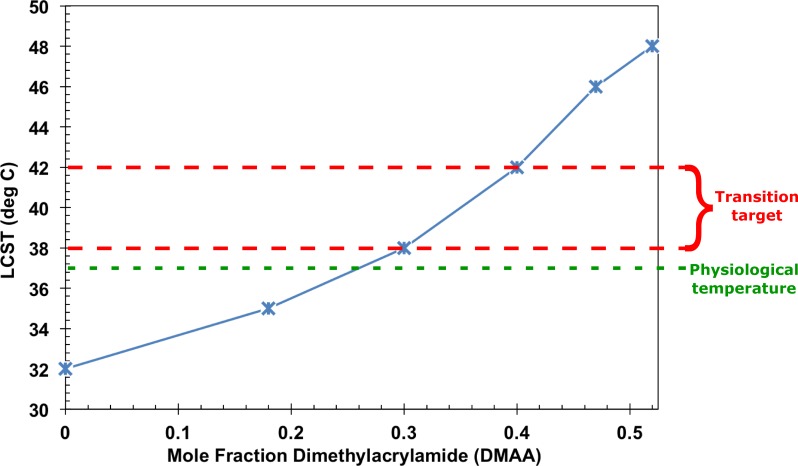
Dimethylacrylamide (DMAA)-dependence of the lower critical solution temperature (LCST) of the copolymer. Datapoints are based on visual determination of solution steady-state opalescence at fixed temperatures. A data connecting line is provided for trend visualization.

Utilizing our approach, we first conducted a preliminary study to establish the thermal transition temperature profile of the pNIPAM-DMAA polymer-valve body formulation as a function of DMAA mole fraction. Resultant LCSTs were estimated by observations of copolymer opalescence at steady state temperatures achieved via temperature regulated water bath immersion. As presented in Fig 3, the LCSTs ranged from 32°C, for 0-mole·fraction DMAA, to 48°C, for 0.52-mole·fraction DMAA. As the primary goal was to achieve a transition temperature slightly above physiological, these initial results directed our further studies to polymer compositions of either 0.30 or 0.40-mole·fraction DMAA that provided a LCST of ~40°C and ~42°C, respectively.

### Compatibility with ethylene oxide (EtO) sterilization

Heater cores with grafted pNIPAM-DMAA of 0.30-mole·fraction DMAA, as described, were extensively dialyzed against distilled water and dehydrated at ambient temperature (~21°C) in a laminar-flow hood. This resulted in collapse of the polymer-valve body against the internal glass core. Collapsed valves were then inserted into individual 1.6 mL Eppendorf tubes and shipped to facilities at Baylor College of Medicine for EtO sterilization. At Baylor, Eppendorf tubes were opened and placed in standard EtO sterilization pouches. A set of three packaged valves then underwent standard EtO processing, while the other set of three packaged valves were handled similarly but without EtO processing.

**Fig 4:**
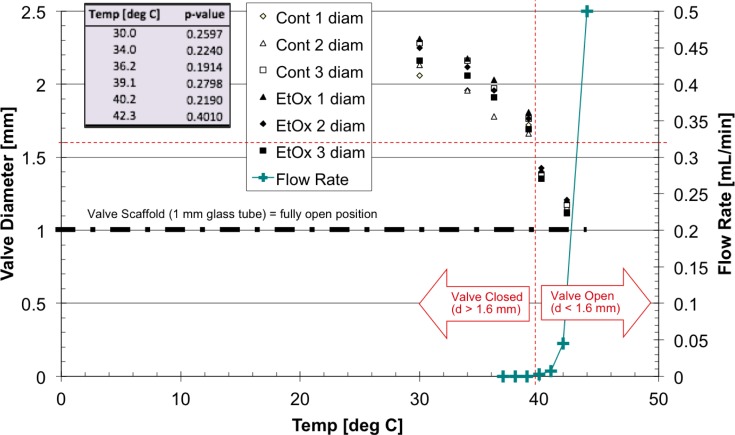
Thermal responsive performance of 30% DMAA pNIPAM valves in caprine amniotic fluid following dehydration, ethylene oxide (EtO) sterilization, then rehydration. P-values of control vs. exposed to EtO sterilization valves indicate that the sterilization procedure does not impact valve performance. ‘Flow Rate’ data (teal color: +) present measured flow rates, based on 80-mmHg hydrostatic head pressure, at various temperatures for a representative valve. Valve diameter data are plotted as black symbols: ■, ☐, ◆, Δ, etc.

Following EtO processing, the valves (3 EtO-processed and 3 controls) were placed under caprine amniotic fluid at 30°C within a microscope dish heating assembly on a Nikon Diaphot 200 microscope. Upon fluid immersion, the collapsed polymer-valve body began to swell and rehydration was complete within ~60 minutes based on microscopic inspection of the polymer-valve diameter. Next, the diameter of each valve was measured from optical micrographs taken at various steady state temperatures ranging from 30–44°C. Fig 4 (left axis) presents the temperature dependence of the valve diameter, comprising a 1-mm diameter glass heater core and its grafted polymer coating, for both the control and EtO treated groups. When installed within a 1.6-mm diameter lumen, this valve formulation would be expected to provide a closed condition under physiological temperatures and to begin opening within the lumen at ~40°C and to complete opening by ~44°C. Student t-tests indicated no statistically significant difference in overall diameter and in the thermal dependence of valve unit operation following EtO sterilization as compared to the untreated controls.

### Steady state flow evaluations

Steady state flow and repeatability of thermo-responsive polymer valves was evaluated by placing valve units within a stirred water bath and measuring the resultant volumetric flow at various bath temperatures under a fixed hydrostatic head pressure. Heater cores with grafted pNIPAM-DMAA, as described, were immersed into a hot water bath (60°C) to collapse the polymer-valve body against the internal glass core. Collapsed valve units were then inserted into and immobilized at the midpoint of 1.78-mm i.d. glass capillaries of 10-cm length by means of a wire tethered through the hollow interior of the heater core. These assemblies were then installed into a flow setup which provided hydrostatic fluid head pressure by variation of the height of a water reservoir above the outlet of the system. The entire assembly was contained within a water-jacketed incubator maintained at 37°C, with the stirred water bath providing additional heating to the capillary element containing the thermo-reactive polymer-valve unit. Prior to flow testing, the manifold was first charged with water by immersing the capillary containing the polymer-valve unit into a 60°C water bath and forcing water through the system with a syringe. The fluid reservoir was then positioned to provide a head pressure of 80-mmH_2_O to simulate sufficient lung growth functional pulmonary fluid pressure of the fetal lamb [31]. Flow measurements were conducted by first setting the water bath at fixed temperatures and allowing the capillary assembly to equilibrate to this temperature over a 30-minute period. Subsequently, measurements were taken at each fixed temperature point by weighing the fluid discharge accumulated over a 2-minute period. This approach was adopted instead of implementation of direct joule heating using the embedded heater element or via external RF signal induction coupling because the thermostatic bath ensured reliable reproducibility and maintenance of valve unit temperature for the rigorous collection of temperature-set-point flow rate datasets.

Fig 4 (right axis) presents flow data from an EtO sterilized valve unit comprised of 0.30-mole·fraction DMAA. At temperatures less than approximately 40°C, the polymer-valve body is swollen and the annular valve unit blocks the principle passage of fluid through the outer valve-containment capillary housing. As temperature is increased above 40°C, the polymer-valve body begins to collapse against its internal glass core allowing for principle flow through the resultant annular gap between the polymer-valve body and the outer valve-containment capillary housing (see Fig 1).

**Fig 5:**
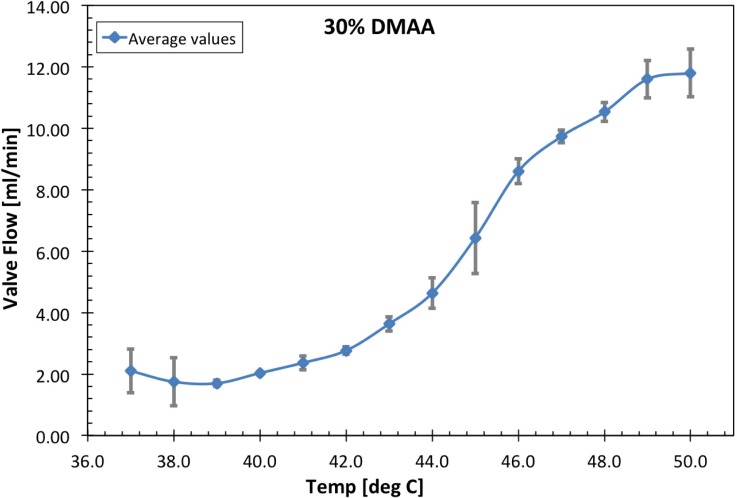
Average temperature dependent flow response performance of 30% DMAA fraction valves with standard deviation bars included and a data connecting line added for trend visualization. These were characterized in caprine amniotic fluid following dehydration, ethylene oxide (EtO) sterilization, then rehydration.

**Fig 6:**
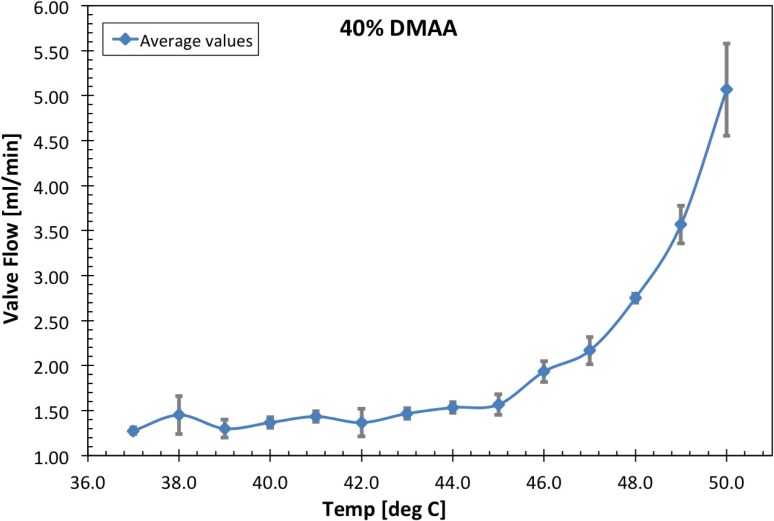
Average temperature dependent flow response performance of 40% DMAA fraction valves with standard deviation bars included and a data connecting line added for trend visualization. These were characterized in caprine amniotic fluid following dehydration, ethylene oxide (EtO) sterilization, then rehydration.

Figs 5 and 6 present the temperature dependent flow responses of grafted pNIPAM-DMAA valves with DMAA content of 0.3 and 0.4-mole·fractions, respectively. Measurements were taken at 1°C increments over a temperature range of 37–50°C and repeated over a period of several days. The plotted values represent the average values measured with corresponding standard deviation bars included. At physiological temperature of 37°C, each valve has a fixed leakage flow rate. This contribution is due to the incomplete and non-uniform sealing of the polymer-valve body against the inner diameter of the outer valve-containment capillary housing in addition to a minor trickle flow through the 0.5-mm i.d. hollow heater-core-capillary upon which the polymer-valve body was grafted. Note that the latter flow component is a consequence of the method by which the valve unit was immobilized within the flow channel. As temperature is increased, the 0.3-mole·fraction DMAA valve unit exhibits increasing flow rate in a sigmoidal fashion with a maximum slope at ~45°C. This flow characteristic is attributed to the temperature-dependent incremental collapse of the polymer-valve body and the ensuing opening of an annular flow path between the polymer-valve body and the surrounding outer valve-containment capillary housing, as depicted in Fig 1. Evidently, the 0.4-mole·fraction DMAA valve unit thermal responses in Fig 6 are a shift to higher temperatures of the flow profile of 0.3-mole·fraction DMAA valve units in Fig 5.

Day to day reproducibility of valve unit flow rates was assessed using the coefficient of variation (*CV* = sigma/mu). The average percent-CV was 11.24% and 6.46% for the data presented in Figs 5 and 6, respectively. The notable variation outliers observed in the initial flow rates at 37°C and 38°C in Fig 5, are predominantly attributed to the accumulation and/or release of air bubbles (i.e., vapor occlusions). These originate from the polymer-valve body itself and/or from within the internal minor flow path through the hollow 0.5-mm i.d. capillary support structure.

## Conclusions

Polymer-valve units comprised of a copolymer of NIPAM and DMAA were constructed that featured phase transition temperatures slightly above physiological body temperature of 37°C. Optimized pNIPAM-DMAA formulation valve units comprising 0.3-mole·fraction DMAA, exhibited phase transitions of ~40°C, maintained their temperature-dependent operation post ethylene oxide sterilization, and performed reproducibly in flow studies conducted over three separate days. These results potentiate further development of this thermo-responsive polymer formulation for clinically-controllable, RF-heating actuatable, implantable polymer valves suitable for applications that include *dynamic* fetal tracheal occlusion therapy.

## Supporting information

S1 Table Data for LCST temperature and DMAA mole-fraction (Fig 3_data). (DOCX)

S2 Table Data for diameter of valves as function of caprine amniotic fluid flow temperature for 3 EtO-processed and 3 controls (Fig 4A_data). (DOCX)

S3 Table P-Value data (Fig 4B_data). (DOCX)

S4 Table Flow Rate data (Fig 4C_data). (DOCX)

S5 Table Data for 30% DMAA valves (Fig 5_data). (DOCX)

S6 Table Data for 40% DMAA valves (Fig 6_data). (DOCX)
