# Supplementary material for: Characterization of a reversible thermally-actuated polymer-valve: A potential dynamic treatment for congenital diaphragmatic hernia
Source: PLoS One. 2018 Dec 27;13(12):e0209855. doi: 10.1371/journal.pone.0209855 (PMC6307748; doi:10.1371/journal.pone.0209855)
Supplement: S1 Table — (DOCX) [file pone.0209855.s001.docx]

**Data for LCST temperature and DMAA mole-fraction**

| DMAA mole-fraction | Temp [deg C] |
| --- | --- |
| 0.00 | 32.0 |
| 0.18 | 35.0 |
| 0.30 | 38.0 |
| 0.40 | 42.0 |
| 0.47 | 46.0 |
| 0.52 | 48.0 |
