# Supplementary material for: Characterization of a reversible thermally-actuated polymer-valve: A potential dynamic treatment for congenital diaphragmatic hernia
Source: PLoS One. 2018 Dec 27;13(12):e0209855. doi: 10.1371/journal.pone.0209855 (PMC6307748; doi:10.1371/journal.pone.0209855)
Supplement: S2 Table — (DOCX) [file pone.0209855.s002.docx]

**Data for diameter of valves as function of caprine amniotic fluid flow temperature for 3 EtO-processed and 3 controls**

| Temp [deg C] | Cont 1 diam | Cont 2 diam | Cont 3 diam | EtOx 1 diam | EtOx 2 diam | EtOx 3 diam |
| --- | --- | --- | --- | --- | --- | --- |
| 30.0 | 2.058823529 | 2.132352941 | 2.279411765 | 2.308823529 | 2.250000000 | 2.161764706 |
| 34.0 | 1.955882353 | 1.955882353 | 2.161764706 | 2.176470588 | 2.117647059 | 2.058823529 |
| 36.2 | 1.911764706 | 1.779411765 | 1.970588235 | 2.029411765 | 1.955882353 | 1.911764706 |
| 39.1 | 1.720588235 | 1.661764706 | 1.764705882 | 1.808823529 | 1.764705882 | 1.691176471 |
| 40.2 | 1.367647059 | 1.352941176 | 1.382352941 | 1.411764706 | 1.426470588 | 1.352941176 |
| 42.3 | 1.161764706 | 1.161764706 | 1.176470588 | 1.205882353 | 1.205882353 | 1.117647059 |

Cont=control valve; EtOx = ethylene oxide (EtO) processed valve; diam = diameter [mm]
