# Supplementary material for: Characterization of a reversible thermally-actuated polymer-valve: A potential dynamic treatment for congenital diaphragmatic hernia
Source: PLoS One. 2018 Dec 27;13(12):e0209855. doi: 10.1371/journal.pone.0209855 (PMC6307748; doi:10.1371/journal.pone.0209855)
Supplement: S3 Table — (DOCX) [file pone.0209855.s003.docx]

**P-Value data**

| Temp [deg C] | p-value |
| --- | --- |
| 30.0 | 0.2597 |
| 34.0 | 0.2240 |
| 36.2 | 0.1914 |
| 39.1 | 0.2798 |
| 40.2 | 0.2190 |
| 42.3 | 0.4010 |
