# Supplementary material for: Characterization of a reversible thermally-actuated polymer-valve: A potential dynamic treatment for congenital diaphragmatic hernia
Source: PLoS One. 2018 Dec 27;13(12):e0209855. doi: 10.1371/journal.pone.0209855 (PMC6307748; doi:10.1371/journal.pone.0209855)
Supplement: S4 Table — (DOCX) [file pone.0209855.s004.docx]

**Flow Rate data**

| Temp [deg C] | Flow [mL/min] |
| --- | --- |
| 37.0 | No Flow |
| 38.0 | No Flow |
| 39.0 | No Flow |
| 40.0 | 0.0025 |
| 41.0 | 0.0075 |
| 42.0 | 0.045 |
| 44.0 | 0.500 |
