# Supplementary material for: Characterization of a reversible thermally-actuated polymer-valve: A potential dynamic treatment for congenital diaphragmatic hernia
Source: PLoS One. 2018 Dec 27;13(12):e0209855. doi: 10.1371/journal.pone.0209855 (PMC6307748; doi:10.1371/journal.pone.0209855)
Supplement: S5 Table — (DOCX) [file pone.0209855.s005.docx]

**Data for 30% DMAA valves**

| Temp [deg C] | Flow Rate [mm/min] | | | | | |
| --- | --- | --- | --- | --- | --- | --- |
|  | Day 1 | Day 2 | Day 3 | Avg. | Std | % CV |
| 37.0 |  | 2.60 | 1.60 | 2.10 | 0.71 | 33.67 |
| 38.0 |  | 2.30 | 1.20 | 1.75 | 0.78 | 44.45 |
| 39.0 | 1.60 | 1.70 | 1.80 | 1.70 | 0.10 | 5.88 |
| 40.0 | 2.00 | 2.10 | 2.00 | 2.03 | 0.06 | 2.84 |
| 41.0 | 2.50 | 2.50 | 2.10 | 2.37 | 0.23 | 9.76 |
| 42.0 | 2.70 | 2.90 | 2.70 | 2.77 | 0.12 | 4.17 |
| 43.0 | 3.90 | 3.50 | 3.50 | 3.63 | 0.23 | 6.36 |
| 44.0 | 5.20 | 4.30 | 4.40 | 4.63 | 0.49 | 10.65 |
| 45.0 | 7.50 | 5.20 | 6.60 | 6.43 | 1.16 | 18.02 |
| 46.0 | 9.00 | 8.20 | 8.60 | 8.60 | 0.40 | 4.65 |
| 47.0 | 9.50 | 9.80 | 9.90 | 9.73 | 0.21 | 2.14 |
| 48.0 | 10.20 | 10.80 | 10.60 | 10.53 | 0.31 | 2.90 |
| 49.0 | 10.90 | 11.90 | 12.00 | 11.60 | 0.61 | 5.24 |
| 50.0 | 10.90 | 12.20 | 12.30 | 11.80 | 0.78 | 6.62 |
| Average % CV | | | | | | 11.24 |

CV=coefficient of variation
