# Supplementary material for: Characterization of a reversible thermally-actuated polymer-valve: A potential dynamic treatment for congenital diaphragmatic hernia
Source: PLoS One. 2018 Dec 27;13(12):e0209855. doi: 10.1371/journal.pone.0209855 (PMC6307748; doi:10.1371/journal.pone.0209855)
Supplement: S6 Table — (DOCX) [file pone.0209855.s006.docx]

**Data for 40% DMAA valves**

| Temp [deg C] | Flow Rate [mm/min] | | | | | |
| --- | --- | --- | --- | --- | --- | --- |
|  | Day 1 | Day 2 | Day 3 | Avg. | Std | % CV |
| 37.0 |  | 1.30 | 1.25 | 1.28 | 0.04 | 2.77 |
| 38.0 |  | 1.30 | 1.60 | 1.45 | 0.21 | 14.63 |
| 39.0 | 1.20 | 1.40 | 1.30 | 1.30 | 0.10 | 7.69 |
| 40.0 | 1.40 | 1.40 | 1.30 | 1.37 | 0.06 | 4.22 |
| 41.0 | 1.40 | 1.50 | 1.40 | 1.43 | 0.06 | 4.03 |
| 42.0 | 1.20 | 1.50 | 1.40 | 1.37 | 0.15 | 11.18 |
| 43.0 | 1.40 | 1.50 | 1.50 | 1.47 | 0.06 | 3.94 |
| 44.0 | 1.50 | 1.60 | 1.50 | 1.53 | 0.06 | 3.77 |
| 45.0 | 1.70 | 1.50 | 1.50 | 1.57 | 0.12 | 7.37 |
| 46.0 | 2.00 | 2.00 | 1.80 | 1.93 | 0.12 | 5.97 |
| 47.0 | 2.20 | 2.30 | 2.00 | 2.17 | 0.15 | 7.05 |
| 48.0 | 2.70 | 2.80 | 2.75 | 2.75 | 0.05 | 1.82 |
| 49.0 | 3.40 | 3.80 | 3.50 | 3.57 | 0.21 | 5.84 |
| 50.0 | 4.50 | 5.50 | 5.20 | 5.07 | 0.51 | 10.13 |
| Average % CV | | | | | | 6.46 |

CV=coefficient of variation
